# Sleep Disturbance and Severe Hydrocephalus in a Normally Behaving Wistar Rat With Traumatic Brain Injury

**DOI:** 10.1089/neur.2022.0090

**Published:** 2023-06-19

**Authors:** Jenni Kyyriäinen, Pedro Andrade, Elina Hämäläinen, Asla Pitkänen

**Affiliations:** A.I. Virtanen Institute for Molecular Sciences, University of Eastern Finland, Kuopio, Finland

**Keywords:** brain injury, case study, electroencephalography, hydrocephalus, lateral fluid-percussion injury, sleep

## Abstract

We report on a case study of a Wistar rat that was investigated in detail because it exhibited no N3 sleep in electroencephalography (EEG) after lateral fluid-percussion injury (FPI)-induced traumatic brain injury (TBI). The rat (#112) belonged to a cohort of 28 adult Wistar rats exposed to lateral FPI. Rats were monitored by continuous video EEG for 30 days to follow-up on the evolution of sleep disturbances. The beam walking test was used to measure post-TBI functional recovery. Severity of the cortical lesion area, total brain volume, and cortical volume were measured from histological brain sections. Rat #112 had a normal body and skull appearance. Its baseline body weight did not differ from that of the rest of the cohort. At baseline, rat #112 crossed the beam in 6.3 sec (score range for the rest of the cohort, 4.7–44.3) and showed no evident slipping of the paws, scoring a 5.3 (score range for the rest of cohort, 4.3–6.0). On day 30 post-TBI, however, rat #112 was the only rat with a score of 0 on the beam. Histological analysis at 30 days post-TBI revealed a small 0.6-mm^2^ post-TBI lesion in the somatosensory cortex (lesion size range for the rest of the cohort, 1.2–10.9). The brain volume of rat #112 was 2-fold larger than the mean volume of the rest of the cohort (1592 vs. 758 mm^3^), the ventricles were remarkably enlarged, and the layered cerebral cortex was very thin. Analysis of the sleep EEG revealed that rat #112 had rapid eye movement sleep and wakefulness, but no N3 sleep, during the 72-h EEG epoch analyzed. This case report demonstrates that brain abnormalities presumably unrelated to the impact-induced cortical lesion, such as presumed pre-existing hydrocephalus, may worsen TBI-induced behavioral and electrographical outcome measures and complicate the assessment of the cause of the abnormalities.

## Introduction

In clinical research, case reports support practice-based evidence to inform clinical research and daily care. In pre-clinical research, published case studies are rare. Reports on outliers in the study cohorts, however, if not excluded from study reports and publications, can lead to novel discoveries and guide experimental practices. Here, we report on a pre-clinical case study of a normally looking and behaving Wistar rat (#112) with traumatic brain injury (TBI) that was included in a video (vEEG) electroencephalography (EEG) monitoring study. The case, rat #112, piqued our interest because its sleep EEG lacked N3 sleep, and the rat exhibited severe behavioral deterioration in the beam walking test despite only a very small TBI-related injury in the primary somatosensory cortex. In the histological analysis, however, rat #112 exhibited a severe, presumably pre-existing ventriculomegaly and a large brain volume.

Hydrocephalus is a condition caused by an accumulation of cerebral spinal fluid attributable to an imbalance in its synthesis or absorption, resulting in the enlargement of the cerebral ventricles.^1^ Clinical symptomatology of hydrocephalus includes gait disturbances and cognitive decline.^2,3^ As in humans, the etiology of hydrocephalus in rodents can vary from genetic cases to various brain lesions.^4–7^ In rodents, the classical phenotypical features of hydrocephalus include a dome-shaped skull and reduced growth rate.^8,9^ Other studies on Sprague-Dawley and Wistar rats, golden hamsters, and a marmoset with hydrocephalus reported a normal phenotypical appearance, normal weight gain, and unimpaired motor performance.^10–13^

We report on a case (rat #112) of an outlier in a study cohort, to inform pre-clinical research that a presumed pre-existing hydrocephalus can contribute to unexpected sleep and behavioral post-TBI outcomes.

## Methods

A detailed description of the materials and methods is presented in the Supplementary Material.

## Results

**FIG. 1.**
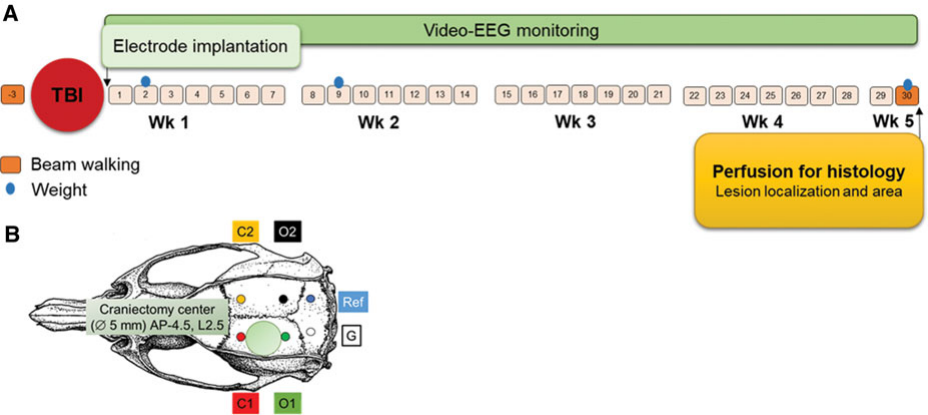
Study design and locations of the recording electrodes. (**A**) TBI was induced in 28 rats, among which 15 completed the 30-day vEEG study. After TBI induction (craniectomy location is shown as a green circle in panel **B**), four epidural screw electrodes (placements in panel [B]: C1, ipsilateral frontal; O1, ipsilateral occipital; C2, contralateral frontal; O2, contralateral occipital) were implanted, and rats were immediately placed under continuous vEEG monitoring for 1 month. Rats were tested in the beam walking test at baseline and on D30 post-TBI. Body weight was recorded on surgery day and then on D2, D9, and D30. Finally, on D31–D34 post-TBI, rats were transcardially perfused for histology, and brains were sectioned and stained with thionin to evaluate the lesion location and area as well as cerebral and cortical volumes. AP, anteroposterior; D, day; EEG, electroencephalography; G, ground; L, lateral; mm, millimeter; Ref, reference; TBI, traumatic brain injury; vEEG, video electroencephalography; Wk, week.

Study design is summarized in Figure 1.

### Narrative of rat #112

Rat #112 flew from Charles-River (UK) to Kuopio (Finland) at the age of 10 weeks with 27 other rats. It had a normal skull and body appearance, and its baseline body weight was comparable to that of the rest of the cohort. It did not show any peculiarities in appearance of the exposed skull or dura during the TBI or electrode implantation surgery. It responded to anesthesia normally. The acquisition of vEEG signal and its quality were comparable to the rest of the cohort. Our suspicions toward “something is abnormal in rat #112” were raised during removal of the perfusion-fixed brain from the skull. The brain appeared “swollen” and transparent (Supplementary Fig. S1C,D). Hydrocephalus became even more apparent during brain sectioning for histology (Supplementary Fig. S1E–H).

### Acute post-impact mortality, exclusions, apnea time, time to righting, occurrence of acute seizure-like behavior, weight, and physiological parameters

**FIG. 2.**
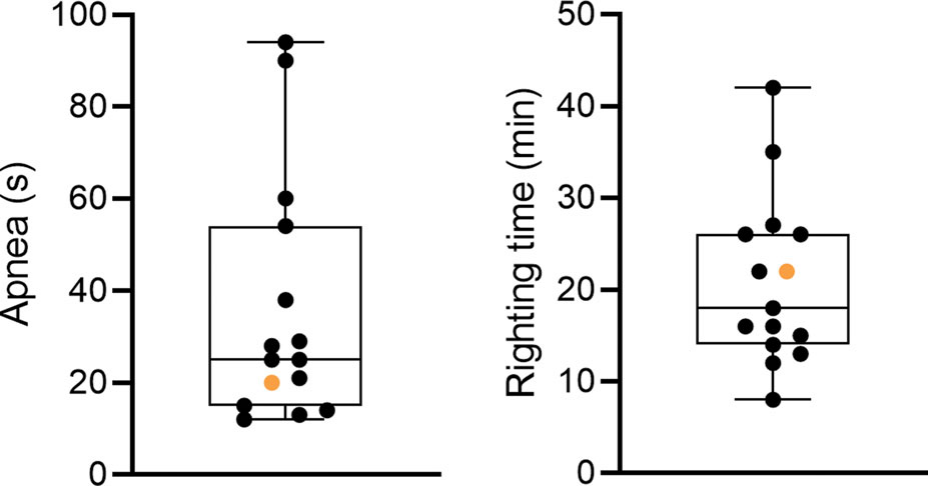
Post-TBI apnea duration (sec) and time to righting (min). (**A**) Apnea time for rat #112 (orange filled circle) was 20.0 sec (rest of cohort mean ± SEM, 37.0 ± 7.3; *n* = 14) and (**B**) time to righting was 22.0 min (rest of cohort mean ± SEM, 20.7 ± 2.5; *n* = 14). Data are presented as whisker plots from minimum to maximum. SEM, standard error of the mean; TBI, traumatic brain injury.

**FIG. 3.**
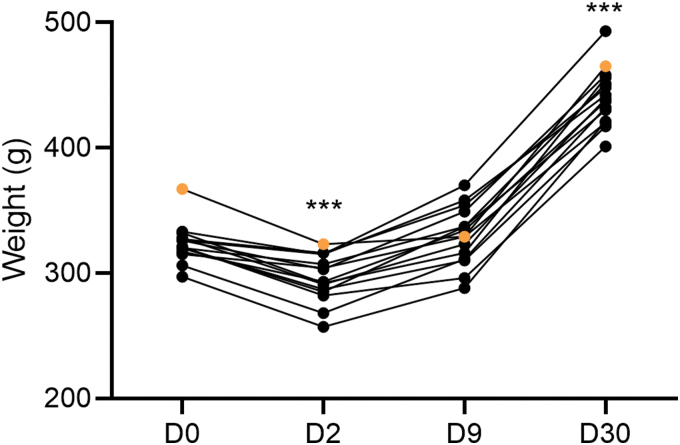
Body weight (g). Body weight of each of the 15 rats over the 30-day follow-up (surgery day [D0], D2, D9, and D30 post-TBI). The weight of rat #112 (orange filled circles) was 367 g on D0, 323 g on D2, 329 g on D9, and 465 g on D30. Statistical significance: ***p < 0.001, compared to D0 (related-samples Friedman's two-way analysis of variance by ranks followed by Wilcoxon's signed-rank test). D, day; TBI, traumatic brain injury.

Data are summarized in Figures 2 and 3 and presented in the text as mean ± standard error of the mean (SEM).

#### Mortality and exclusions

Of the 28 rats, 1 rat was excluded because of a low impact pressure (1 of 28; 4%), 1 rat was found dead on day (D) 1 post-TBI (1 of 28; 4%), and 1 rat died after electrode implantation (1 of 28; 4%). Acute impact-related mortality was 36% (10 of 28 rats). Altogether, 15 rats completed the 30-day follow-up.

#### Apnea and righting reflex

Post-impact apnea time for rat #112 was 20.0 sec (rest of cohort, 37.0 ± 7.3; median, 26.5; range, 12.0–94.0), and time to righting reflex was 22.0 min (rest of cohort, 20.7 ± 2.5; median, 17.0; range, 8.0–42.0; Fig. 2).

#### Acute post-impact seizure-like behavior

Acute post-impact seizure-like behavior was observed in 1 of 15 rats included in the analysis (not rat #112).

#### Body weight

On D0 (day of injury), the body weight of rat #112 was 367 g, the highest within the cohort (rest of cohort, 321 ± 3; median, 321; range, 297–333; Fig. 3). On D2, its body weight was 323 g (rest of cohort, 294 ± 5; median, 293; range, 257–316); on D9, body weight was 329 g (rest of cohort, 329 ± 6; median, 332; range, 288–370), and on D30, body weight was 465 g (rest of cohort, 439 ± 6; median, 438; range, 401–493). The body weight of rat #112 decreased by 12% between D0 and D2 (rest of cohort mean, 8), increasing 2% between D2 and D9 (rest of cohort mean, 12) and 29% between D9 and D30 (rest of cohort mean, 25).

#### Physiological parameters

Before TBI induction, rat #112 had an arterial O_2_ saturation of 92% (rest of cohort, 91 ± 2; median, 90; range, 86–100; *n* = 8), heart rate (beats per minute) of 611 (rest of cohort, 553 ± 59; median, 540; range, 367–775; *n* = 8), pulse distention (μm) of 10 (rest of cohort, 34 ± 14; median, 20; range, 11–130; *n* = 8), breathing distention (μm) 31 (rest of cohort, 42 ± 24; median, 21; range, 5–207; *n* = 8), and breathing rate (breaths per minute) of 331 (rest of cohort, 209 ± 53; median, 216; range, 41–410; *n* = 8). No post-TBI physiological parameters were available for rat #112.

### Beam walking

Data are summarized in Supplementary Figures S2 and S4 and in Supplementary Videos S1 and S2 and presented in the text as mean ± SEM.

**FIG. 4.**
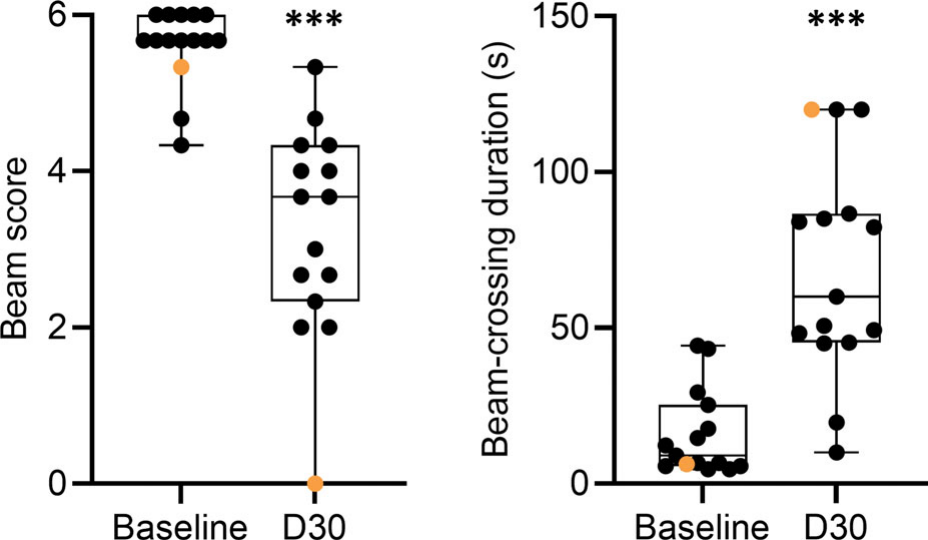
Beam walking. (**A**) Baseline beam score for rat #112 (orange filled circle) was 5.3 (rest of cohort mean ± SEM, 5.6 ± 0.1). On D30 post-TBI, rat #112 was the only rat with a score of 0 on the beam. (**B**) At baseline, rat #112 needed 6.3 sec (rest of cohort mean ± SEM, 16.4 ± 3.7) to cross the beam. On D30, rat #112 showed a complete loss of balance. Statistical significance: ***p < 0.001, compared with the corresponding baseline value (Wilcoxon's signed-rank test). Data are presented as whisker plots from minimum to maximum. D, day; SEM, standard error of the mean; TBI, traumatic brain injury.

Physical appearance, skull shape, and skull size of rat #112 appeared normal (Supplementary Fig. S2; Supplementary Video S1). At baseline, rat #112 exhibited normal motor balance given that it crossed the beam with a minimal number of paw slips and scored 5.3 (rest of cohort, 5.6 ± 0.1; median, 5.7; range, 4.3–6.0; Fig. 4; Supplementary Video S1). Also, the beam-crossing duration of rat #112 was 6.3 sec, which was one of the faster times of the cohort (rest of cohort, 16.4 ± 3.7; median, 10.7; range, 4.7–44.3). On D30 post-TBI, however, rat #112 was the only rat of the cohort that scored 0.0 on the beam (rest of cohort, 3.5 ± 0.3; median, 3.7; range, 2.0–5.3), indicating a complete loss of balance and inability to cross the beam (Supplementary Video S2). Consequently, beam-crossing duration was assigned a maximum of 120.0 sec (rest of cohort, 64.7 ± 8.8; median, 55.3; range, 10.0–120.0).

### Cerebral gross anatomy, brain and cortical volumes, and cortical lesion area

**FIG. 5.**
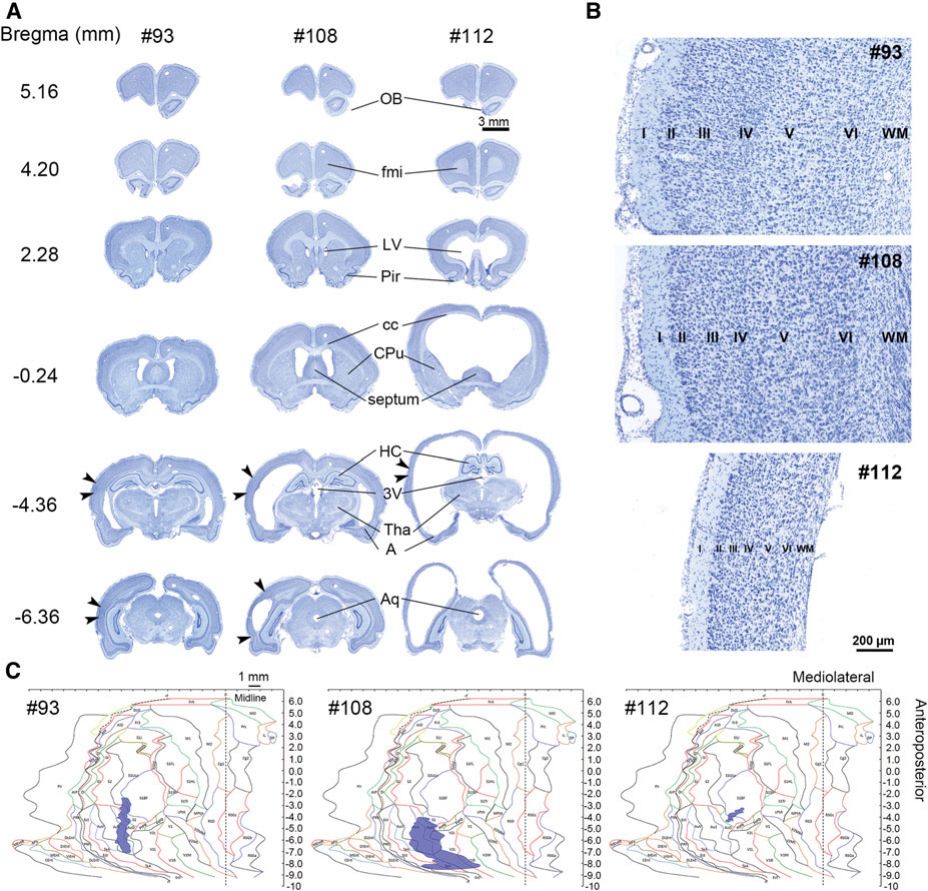
Gross anatomy and location and area (mm^2^) of the cortical lesion. (**A**) Panels show representative thionin-stained coronal sections of the rat with a small (left, #93) or large lesion (middle, #108) and rat #112 (right) on D31–D34 post-TBI. Arrowheads indicate the mediolateral lesion extent at different bregma levels (bregma 5.16 to −6.36). Note the enlarged ventricles and robustly reduced cortical thickness in rat #112. Also, the septal region, putamen, and rostral hippocampus were compressed at the bottom of the cranium. (**B**) Panels show the cortical lamination in each of the three cases (rats #93, #108, and #112). Scale bar is the same for all cases, illustrating the dramatic thinning of the cerebral cortex in rat #112. (**C**) Panel shows computer-generated unfolded cortical maps, indicating the lesion location on the cortical mantle (violet) in each animal. Rat #112 had a small (0.6 mm^2^) post-TBI lesion in the somatosensory cortex (rest of cohort range, 1.2–10.9). 3V, third ventricle; Aq, aqueduct; A, amygdala; cc, corpus callosum; CPu, caudate putamen (striatum); D, day; fmi, forceps minor corpus callosum; HC, hippocampus; LV, lateral ventricle; OB, olfactory bulb; Pir, piriform cortex; SEM, standard error of the mean; TBI, traumatic brain injury; Tha, thalamus.

**FIG. 6.**
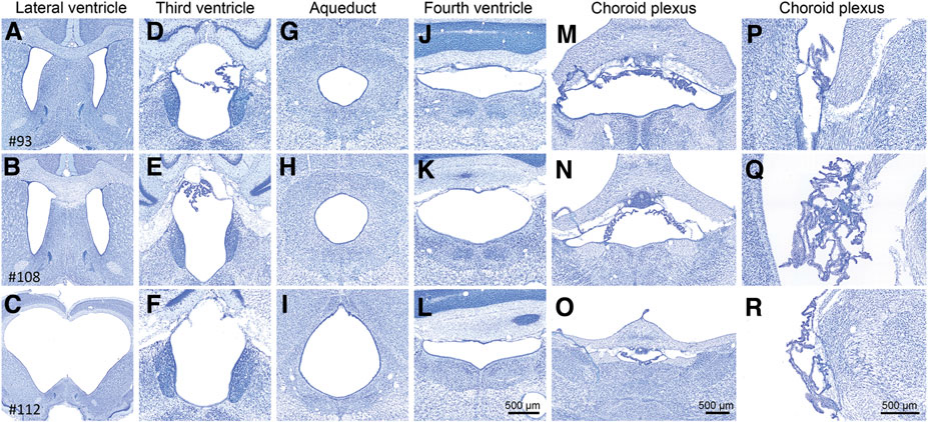
Histological assessment of the cerebrospinal fluid pathway. Representative thionin-stained coronal sections showing lateral ventricles, third ventricle, aqueduct, fourth ventricle, and choroid plexus of a rat with a small (upper panels, #93) or large cortical lesion (mid panels, #108) and hydrocephalus in rat #112 (lower panels). Despite ventriculomegaly in rat #112, the cerebrospinal fluid pathway was open and comparable to that of the other rats in the TBI cohort. Visually, no hypertrophy or swelling of the choroid plexus was observed. Scale bar in panel L is for panels **A–L**, scale bar in panel O is for panels **M–O**, and scale bar in panel R is for panels **P–R**. TBI, traumatic brain injury.

**FIG. 7.**
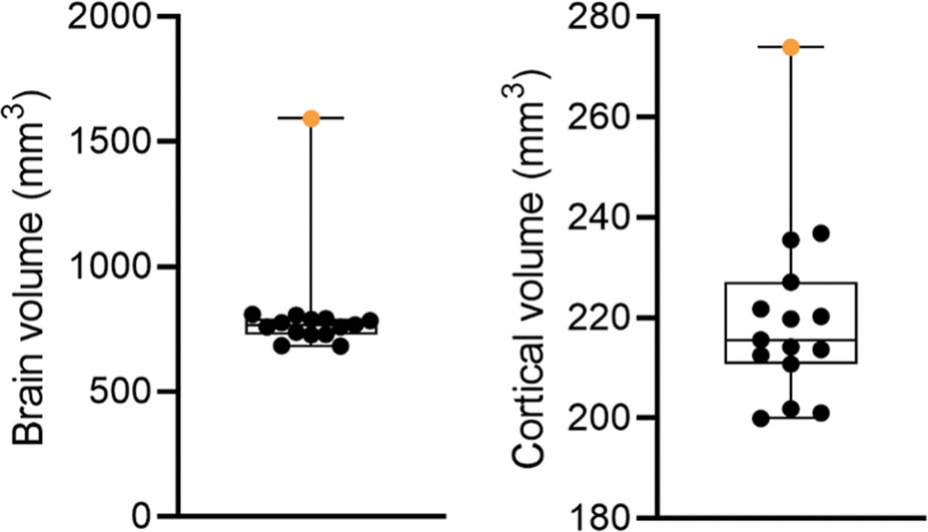
Brain and cortical volumes. The graph shows the total brain volume and cortical volume (mm^3^) of rat #112 (orange filled circle) and the remaining 14 TBI-induced rats in the cohort. The brain volume of rat #112 was 210% of the cohort mean. The cortical volume of rat #112 was 127% of the cohort mean. Data are presented as whisker plots from minimum to maximum. TBI, traumatic brain injury.

Data are summarized in Figures 5–7 and presented in the text as mean ± SEM. Ventricle enlargement, particularly ipsilaterally, is common after moderate-to-severe TBI, as shown in Figures 5 and 6.

#### Brain gross anatomy of rat #112

In visual analysis, the frontal and piriform cortex as well as the olfactory bulbs appeared normal. Rostrally, the lateral ventricles invaded the white matter of the forceps minor of corpus callosum bilaterally. More caudally, there was a remarkable symmetrical enlargement of the lateral ventricles. However, the other areas of the cerebrospinal fluid pathway, that is, the choroid plexus and the volumes of the third and fourth ventricles, appeared comparable to the rest of the TBI cohort (Fig. 6). No obstruction was found in the aqueduct. Iron deposits were found in the choroid plexus in all rats with TBI, including rat #112 (data not shown). Rat #112 also showed a remarkable thinning of the cerebral cortex (particularly caudally) and corpus callosum and detachment of the septum and septal hippocampus from the corpus callosum. Striatum and caudal aspects of the amygdaloid complex, and also the thalamus, were bilaterally atrophied. The septal hippocampus was oedemic bilaterally. However, the internal structure of the dentate and hippocampal cellular layers appeared normal throughout the septotemporal axis. For example, we did not observe neuronal loss in the CA3a region, typical to lateral fluid-percussion injury (FPI)-induced hippocampal injury. The brainstem appeared normal in sections available for analysis.

#### Brain and cortical volumes

The neuroanatomy of two typical post-TBI cases (rats #93 and #108) is shown along with that of rat #112 in the thionin-stained coronal sections of Figure 5A. Brain volume of rat #112 (1592 mm^3^) was 2.1-fold that of the cohort mean (rest of cohort, 758 ± 11 mm^3^; median, 763; range, 683–810; Fig. 7). Cortical volume of rat #112 was 127% (274 mm^3^) that of the cohort mean (rest of cohort, 217 ± 3 mm^3^; median, 215; range, 200–237). Further visual analysis indicated a robustly reduced cortical thickness in rat #112, but cortical lamination was identifiable (Fig. 5B).

#### Post–traumatic brain injury unfolded maps and the cortical lesion

Unfolded cortical maps in Figure 5 demonstrate the location and extent of the cortical lesion in a rat with a small lesion area (rat #93; total area, 3.5 mm^2^), a rat with a large lesion area (rat #108; total area, 10.9 mm^2^), and in rat #112 (total area 0.6 mm^2^ vs. the rest of cohort, 3.9 ± 0.7; median, 3.0; range, 1.2–10.9). Typically, the lesion epicenter was located in the auditory cortex, extending to the somatosensory and visual cortices. In rat #112, the lesion was in the primary somatosensory cortex (S1; 12.7% of S1 area vs. rest of cohort, 17.1 ± 2.5; median, 16.9; range, 3.1–34.5) and in the barrel field of the primary somatosensory cortex (S1BF; 3.7% of S1BF area vs. rest of cohort, 4.3 ± 1.5; median, 2.8; range, 0.7–10.4). The Pearson correlation test showed no association between lesion coverage of the S1, S1BF, or the combined area (S1 + S1BF) and the beam score or the duration of beam crossing at D30 post-TBI (*p* > 0.05).

### Post–traumatic brain injury electroencephalography sleep pattern

**FIG. 8.**
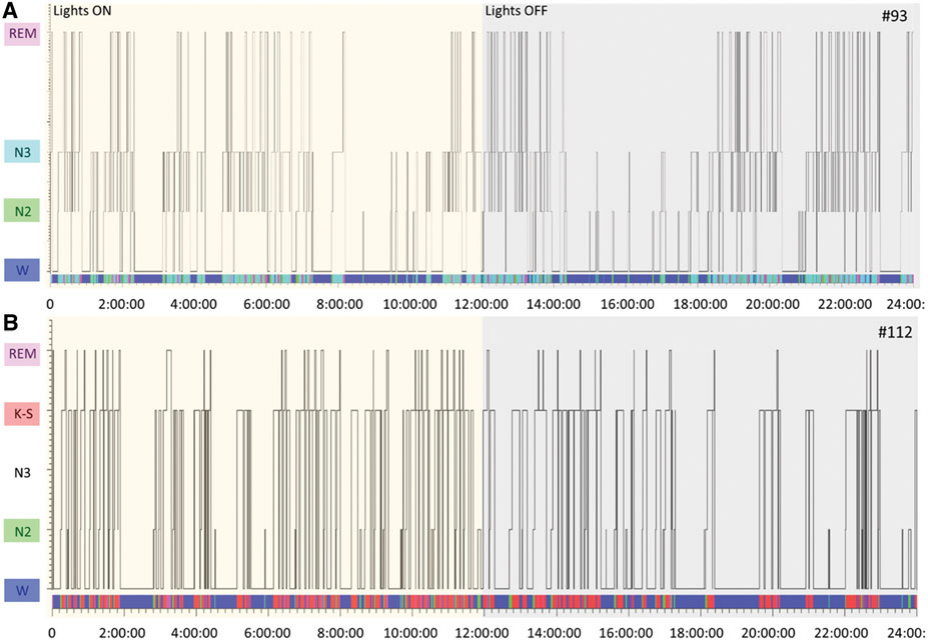
Hypnogram of post-TBI EEG sleep patterns in rats #93 and #112. Hypnogram shows the occurrence of different sleep-wave phase patterns (x-axis) in rats (**A**) #93 and (**B**) #112 during the 24-h EEG epoch (y-axis). EEG activity of rat #112 was dominated by symmetric successive K-spindle complexes. Rat #112 had REM sleep and wakefulness, but no N3 sleep, during the 24-h EEG epoch analyzed. EEG, electroencephalography; K-S, K-spindle; N2; N2 sleep; N3, N3 sleep; REM, rapid eye movement; SEM, standard error of the mean; TBI, traumatic brain injury; W, wake.

**FIG. 9.**
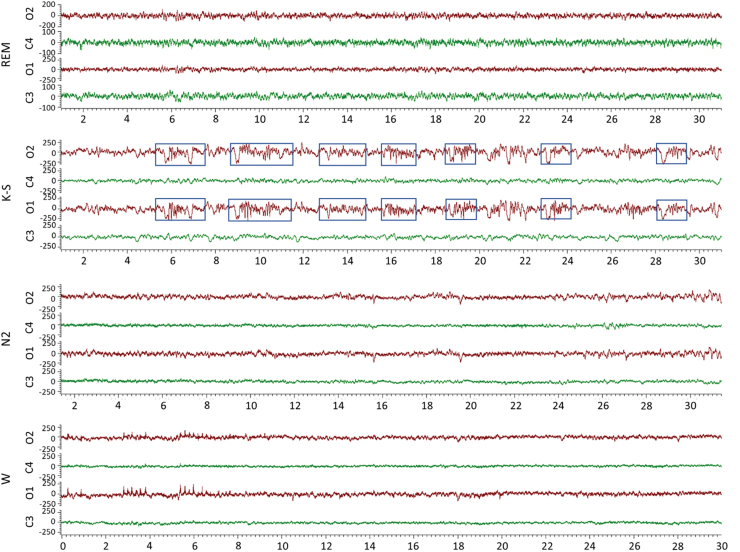
Sleep patterns. EEG (low-frequency filter, 0.5 Hz; high-frequency filter, 100 Hz) showing 30-sec epochs (x-axis) of the typical sleep EEG patterns in rat #112. Note the different y-axis scale in the REM-panel and the occurrence of symmetric bilateral K-spindle complexes (marked with blue boxes) in the K-spindle-panel. C1, ipsilateral frontal; C2, contralateral frontal; EEG, electroencephalography; K-S, K-spindle; N2, N2 sleep; N3, N3 sleep; O1, ipsilateral occipital; O2, contralateral occipital; REM, rapid eye movement; s, second; TBI, traumatic brain injury; W, wake.

During the 30-day vEEG period, rat #112 exhibited suppressed EEG (Supplementary Video S3). As shown in the hypnogram, rat #93 spent 44% of the time awake (10 h 37.5 min), 12% in N2 (2 h 45 min), 34% in N3 (8 h 11 min), and 10% in rapid eye movement (REM; 2 h 27 min) over the 24-h analysis period (Supplementary Video S4). Rat #112 spent 52% of the time awake (12 h 32.5 min), 6% in N2 (1 h 22.5 min), 0% in N3 (0 sec), 37% in the K-spindle phase (K-S; 9 h), and 5% in REM (1 h 5.5 min) over the 24-h analysis period (Figs. 8 and 9). The K-S was dominated by symmetric successive K-spindle complexes (Fig. 9). Patterns were comparable over the entire 72-h analysis period starting on day 11 post-TBI. Analysis on other days, including the first post-injury week, and every 6 days thereafter until 30 days after TBI, also showed that rat #112 had no N3 sleep.

## Discussion

We report on the case of rat #112, which underwent TBI induced with lateral FPI and exhibited ventriculomegaly, large brain volume, severe sleep disturbance with a lack of N3 sleep, and rapid behavioral deterioration post-TBI.

### Rat #112 exhibited a severe sleep disturbance in electroencephalography

Disturbance in the sleep-wake cycle is common after experimental and human TBI.^14^ Previous studies revealed that the thalamus, cerebral cortex, and cortico-thalamo-cortical circuit are necessary for generating sleep.^14–16^ Rat #112 exhibited a severe sleep disturbance with suppressed EEG patterns, reoccurring K-spindle complexes, lack of N3 sleep, a minimal amount of REM sleep, and a notable amount of time spent awake. These sleep features of rat #112 conspicuously distinguished it from all other rats in the cohort. The lack of deep restorative N3 sleep typically results in cycling back to a lighter sleep stage to reinduce a proper N3 stage or in entering straight into REM sleep. To date, only a few reports have described disturbed N3 sleep after TBI.^17,18–21^ Further studies are needed to explore the contribution of hydrocephalus and accompanying pathologies, particularly in sleep-network–related white matter tracts, to clarify the link between hydrocephalus and sleep disturbance.

### Rat #112 exhibited ventriculomegaly, large brain volume, and thin cerebral cortex

Before TBI, rat #112 had a normal physical appearance, skull shape, body weight, and performance in the beam walking test. At 1 month, the area cortical damage induced by lateral FPI was small, being only 0.6 mm^2^. The thinned cortex and enlarged fluid-filled ventricles may have dampened the net effect of the 2.5-atm impact force applied to rat #112, reducing the lesion area. Moreover, even though the skull volume appeared normal, the total volume of the perfusion-fixed cerebrum was 2.1-fold greater than the cohort mean. Consequently, one can speculate that the intraskull movement of the brain exposed to fluid-percussion impact was restricted in rat #112 as compared to that in other rats, reducing the lesion area.

We assume that the hydrocephalus and enlarged brain volume were not TBI related. Rather, they were pre-existing, resembling a case recently reported on by Ferris and colleagues, who reported a 1.8-fold increase in brain volume of a 2-year-old R222 RNaseT2 knockout rat with severe “spontaneous hydrocephalus.”^22^ The etiology of the “pre-existing” hydrocephalus (e.g., genetic) in rat #112 is unknown; another question is how a brain twice the volume of the normal brain could fit into the regular-sized skull. Ferris and colleagues did not report any abnormalities in skull size or shape either, even though the skull appears larger than normal in magnetic resonance imaging (MRI) shown in their Figure 1.^22^ Because we did not perform any structural MRI, we cannot conclude whether the thin cortex of rat #112 was folded within the skull or whether the skull was thinner, giving the brain more space.

Hydrocephalus can be defined as a pathological increase in cerebral ventricular volume caused by impaired absorption or increased production of cerebrospinal fluid.^1,3,23^ Edwards and colleagues identified hydrocephalus in 31 of 35 hamsters without a dome-shaped skull.^10^ The lack of doming suggested that the condition in the hamster cohort developed after suture closure. In another study, hydrocephalic Wistar rats displayed abnormalities in the ventricular and vascular systems, including aneurysms, intracranial cysts, white matter injury, tissue necrosis, and excessive astrogliosis surrounding the ventricles and blood vessels.^11,24^ We observed no doming of the skull in rat #112; nor did we observe any compression of the ventricular system or the Sylvian aqueduct. Iron deposits in the choroid plexus, which were recently associated with post-TBI ventricle enlargement, were detected in all rats of the cohort with TBI.^7^ Therefore, we consider post-injury choroidal hemorrhage as an unlikely etiological factor for the robust ventriculomegaly in rat #112.

Gross anatomical analysis of the brain of rat #112 revealed a very thin cerebral cortex. Contrary to our expectations, the cortical volume of rat #112 was 127% that of the cohort mean. We have compared the cortical volume rat #112 to other TBI-induced rats in the same cohort, rather than to naïve or sham-operated control rats. Further studies are needed to explore whether the cortical volume increase is related to intracortical edema and/or gliosis or simply to stretching of the cortical tissue surrounding the enlarged ventricles. Interestingly, Ferris and colleagues reported a 7% reduction in cortical volume in the R222 RNaseT2 KO rat with severe spontaneous hydrocephalus.^22^

### Rat #112 exhibited greatly impaired motor performance with only a very small post–traumatic brain injury cortical lesion

The pre-injury performance of rat #112 in the beam walking test was normal given that it scored 5.3 of 6. Consistent with this finding, other laboratories have reported minimal behavioral impairments in uninjured hydrocephalic Sprague-Dawley rats or golden hamsters.^10,13^ Tu and colleagues found no phenotypical signs of hydrocephalus in uninjured adult male and female Wistar rats, among which 43% had mild ventriculomegaly in MRI.^11,24^ Also, the 2-year-old R222 RNaseT2 KO hydrocephalus case reported by Ferris and colleagues appeared to have normal sensory behavior, given that it responded normally to foot shock and flashing light and startled to a loud sound.^22^

Interestingly, a minimal cortical lesion induced by FPI resulted in severe impairment in the beam walking test within the 30-day follow-up given that the performance score dropped from 5.3 to 0, which was greater than the mean 2-points drop in the rest of the TBI cohort. Moreover, the lesion was located in the somatosensory cortex (rather than in the auditory cortex), which could explain the behavioral deficits. However, we did not notice any correlation between lesion coverage of the S1 cortices and performance in beam walking, suggesting that the motor impairment related more to hydrocephalus rather than cortical damage. Interestingly, in a model of acquired hydrocephalus induced by an intraventricular kaolin injection, slow motor and cognitive deterioration were reported over an 8-month follow-up, possibly related to stretching of the periventricular axonal pathways because of an increased ventricle volume.^25^

Our observations in rat #112 suggest that the rapid, severe post-impact balance impairment triggered by minimal injury to the sensory cortex was augmented by pre-existing hydrocephalus.

## Conclusions and Lessons Learned

We report on a Wistar rat with a rapid post-injury deterioration in the beam walking test and a lack of N3 sleep despite a very small TBI-related lesion in the somatosensory cortex. Histological analysis revealed severe ventriculomegaly and 2.1-fold enlarged brain volume, which were presumably present before the TBI. Our observations of this case suggest that, although rare, pre-existing hydrocephalus may augment, and consequently complicate, the interpretation of TBI-induced behavioral and electrographical outcome measures. Future studies are needed to explore the effect of congenital and/or acquired hydrocephalus on post-TBI outcome.

To increase the rigor in pre-clinical data management and reporting, the present study provided some lessons to be learned. At baseline, it is important to pay attention and document whether there are any peculiarities in the physical appearance of the skull or in the animal's behavior such as gait. After exposure of the skull during surgery, its shape and size should be noted. During histological processing of the brain, gross assessment of the ventricular size and cortical thickness should be noted. Given that structural MRI is becoming more available, affordable, and faster, in some cases the baseline MRI-based screening of hydrocephalus (or other brain abnormalities) could be considered.

## Supplementary Material

Supplemental data

Supplemental data

Supplemental data

Supplemental data

Supplemental data

Supplemental data

Supplemental data

## Abbreviations Used

D: day
EEG: electroencephalography
FPI: fluid-percussion injury
MRI: magnetic resonance imaging
REM: rapid eye movement
SEM: standard error of the mean
TBI: traumatic brain injury
vEEG: video electroencephalography
