# Supplementary material for: Sleep Disturbance and Severe Hydrocephalus in a Normally Behaving Wistar Rat With Traumatic Brain Injury
Source: Neurotrauma Rep. 2023 Jun 19;4(1):384–95. doi: 10.1089/neur.2022.0090 (PMC10282974; doi:10.1089/neur.2022.0090)
Supplement: Supplemental data [file Suppl_Material.docx]

**Sleep Disturbance and Severe Hydrocephalus in a Normally Behaving Wistar Rat with Traumatic Brain Injury**

*Jenni Kyyriäinen^1^, Pedro Andrade^1^, Elina Hämäläinen^1^, Asla Pitkänen^1^*

*^1^A. I. Virtanen Institute for Molecular Sciences,*

*University of Eastern Finland, PO Box 1627, FI-70211 Kuopio, Finland*

**Corresponding author:** Asla Pitkänen, MD, PhD, A.I. Virtanen Institute for Molecular Sciences, University of Eastern Finland, PO Box 1627, FI-70211 Kuopio, Finland, Tel: +358-50-517 2091, E-mail: [asla.pitkanen@uef.fi](about:blank)

**Key words:** brain injury - case study - electroencephalography - hydrocephalus - lateral fluid percussion injury - sleep

**Running title:** Hydrocephalus and TBI in a Wistar rat

**SUPPLEMENTARY FILES**

**Materials and methods**

**Rats**

Adult (12 wk-old) male Wistar (strain code 003, Charles River, UK) rats (n = 28) were single-housed (cage size: 27.4 x 44.8 x 23.2 cm) in a controlled environment (temperature 21 ± 2°C, humidity 55 ± 15%, light-dark cycle from 07.00-19.00) with free access to food (Teklad 2018S) and water. Rats arrived in quarantine 1 week before the baseline behavioral testing (beam walking test); that is, 2 weeks prior to TBI induction. All animal procedures were approved by the Animal Ethics Committee of the Provincial Government of Southern Finland and performed in accordance with the guidelines of the European Community Council Directives 2010/63/EU.

**Lateral fluid-percussion injury (FPI)**

TBI was induced by lateral FPI according to a slightly modified protocol of McIntosh et al.^26^ The rat was anesthetized with isoflurane (5% induction, 2.2-2.7% maintenance, air 500 ml/min, Kent Scientific SomnoSuite®), and fixed to a stereotaxic frame using blunt ear bars. Lidocaine (10 mg/ml, volume 0.1 ml) was injected subcutaneously (s.c.) into the skin over the planned craniectomy. The rat was connected to MouseOx® Plus (STARR Life Sciences, rat sensors) to monitor the pre-injury heart rate (bpm), breathing rate (brpm), arterial oxygen saturation (%), pulse distention (µm), and breathing distention (µm). A midline incision was made, the skin and temporal muscles were reflected, and a 5-mm craniectomy was created over the left cortex with hand-held trephine midway between lambda and bregma, and midway between the sagittal suture and temporal ridge (craniectomy center: AP -4.5 mm; ML 2.5 mm).^27^ The intactness of the dura was checked before placing a plastic female-female Luer-lock injury cap (Cole-Palmer; #4550-22) around the edges of the craniectomy, and sealing it to the skull with 3M tissue glue (3M, St. Paul, MN, USA). The outside of the injury cap was further sealed with cyanoacrylate (Loctite®). The injury cap was then filled with sterile 0.9% sodium chloride (NaCl) and further stabilized with dental cement (Selectaplus, DeguDent GmbH, Germany). The rat was then removed from the stereotaxic frame and MouseOx® Plus and placed on its ventral surface. The rat was immediately connected to the curved tip of the FPI device (AmScien Instruments, Richmond, Virginia, USA, model 302) via a screw-lock connector attached to braided pressure tubing (2 mm diameter, 16 cm long). Time to respond to a left hind paw toe pinch was recorded. A pressure pulse (mean 2.42 ± 0.05 atm) was delivered immediately after the rat responded to a toe pinch and its severity was monitored with a PC-based automatic pressure measurement unit. Duration of post-impact apnea and occurrence of post-impact seizure-like behavior were monitored. The rat was then moved onto a heating pad (38°C) and turned on its right side, its tongue was pulled out to facilitate breathing, the rat was reconnected to the MouseOx® Plus, and supplementary oxygen was administered via a nose mask. Time to righting (min) was measured. The rat was then reanesthetized with 5% isoflurane (maintenance 2.2-2.7%) and the injury cap was removed.

**Electrode implantation for EEG recordings**

Immediately after removing the injury cap, the rats were placed back into the stereotaxic frame. Four stainless steel epidural screw EEG recording electrodes (0.5 mm, EM12/20/SPC, P1 Technologies, Roanoke, VA, USA), 2 ipsilateral and 2 contralateral, were placed over the frontal cortex (**C1:** AP -1.7, ML, left 2.5; **C2:** AP -1.7, ML, right -2.5) and the parieto-occipital cortex (**O1:** AP -7.6, ML, left 2.5; **O2:** AP -7.6, ML, right -2.5). In addition, 2 electrodes were inserted into the skull bilaterally over the cerebellum to serve as reference and ground electrodes (see **Fig. 1**). The electrodes were attached to a multi-pin connector (MS12P, P1 Technologies), according to a monopolar referential montage. Electrodes were fixed to the skull using dental cement (Selectaplus). Lastly, buprenorphine (INDIVIOR, 0.3 mg/ml, volume 0.2 ml) and 10 ml of 0.9% NaCl were administered (s.c.).

**Animal monitoring and care during and after operation**

Bodyweight was recorded on the day of injury, and again on day (D) 2, D9, and D30 post-TBI (Scale 1, #PT1500; Scale 2, #BP3100; SARTORIUM Vendor).

After TBI, the rats were fed normal Teklad 2018S pellets and supplementary food (Hills a/d for 3 d). In addition, 20 ml of 0.9% NaCl (s.c.) per day for 3 d was administered for supplementary volume correction.

**Video-EEG monitoring**

Continuous video-EEG monitoring lasting 30 d was started immediately after induction of TBI and electrode implantation. For monitoring, rats were single-housed in Plexiglas cages (44 [length] x 29 [width] x 50 [height] cm) and connected to an amplifier via cables (M12C-363/2, Plastic P1 Technologies) and 6-pin commutators (SL12C), allowing free movement. EEG was monitored using the Nicolet One EEG (ver. 5.71) recording system connected to a M40 (Taugagreining, Iceland) or Oxford (Medical Systems Division, UK) amplifiers and filtered (high-pass filter 0.3 Hz, low-pass 100 Hz). The behavior of each rat was recorded using a cage-specific 1.2 MP resolution camera (Basler DAA1280-54UM/S, 30 frames/second) that was positioned behind the cage. Cage-specific infrared illumination (24 V, 150 mA) was used at night to allow continuous 24/7 video-monitoring. Synchronization of the video and EEG files within 0.1 s was achieved using in-house software that creates a metafile with a precise timestamp for both the EEG and the video. For data storage, the video-EEG system was connected to a 35 TB NAS configured to RAID6 for data redundancy.

**Analysis of sleep in video-EEG recordings**

Each video-EEG raw data file was converted into 24 bits, and imported to Spike2 (version 9, CED, UK). The sleep stages were manually scored from a 72-h–long recording, starting on D11 post-TBI. Sleep scoring was performed according to the American Academy of Sleep Medicine (2007) guidelines with some adaptations to rat sleep-EEG described previously.^17^

**Behavioral analysis**

The beam walking test was performed at baseline and on D30 post-TBI to evaluate the injury effect on complex motor movement and coordination.^28^ Briefly, rats were habituated to the beam (1390 mm long, 21 mm wide wooden bar placed 430 mm above the floor) and a black box (250 x 200 mm located at the end of the beam). On the testing day, rats were allowed to walk the beam 3 times and to remain in the black box for 1 min between each of the 3 test runs. Behavior on the beam was scored from 0 (falls from the beam) to 6 (walks the entire beam without slipping), and the mean score for the 3 runs was calculated. As other outcome measures, we recorded the time to walk the beam and the mean walking duration of 3 runs was calculated. If the rat remained sitting on the beam (score 1), or fell down immediately (score 0) or before crossing the beam completely (score 2), it was assigned a time 120 s.

**Histology**

To assess cortical lesion location and area, rats were perfused for histology at D31–34 post-TBI.

***Fixation.*** Rats were deeply anesthetized with an intraperitoneal injection of sodium pentobarbital (60 mg/kg) and intracardially perfused with 0.9% NaCl (6 min, 30 ml/min) followed by 4% paraformaldehyde (30 min, 30 ml/min). The brains were removed from the skull, fixed in 4% paraformaldehyde for 4 h, cryoprotected in 20% glycerol in 0.02 M potassium phosphate buffer (pH 7.4 for 24 h), frozen in dry ice, and stored at -70°C for further processing. Frozen coronal sections were cut (30-µm thick, 1-in-5 series) using a sliding microtome.^29^ The first series stored in 10% formalin at room temperature was used for thionin staining. The remaining series of sections were collected into tissue collection solution (30% ethylene glycol, 25% glycerol in 0.05 M sodium phosphate buffer) and stored at -20°C until processed.

***Nissl staining.*** The first series of sections was stained with thionin, cleared in xylene, and cover-slipped using Depex® (BDH Chemical, Poole, UK) as a mounting medium.

***Unfolded cortical maps.*** To assess the severity and cytoarchitectonic distribution of the lesion extent after TBI, all thionin-stained sections were digitized (40x, Hamamatsu Photonics, NanoZoomer-XR, NDP.scan 3.2). Unfolded cortical maps were then prepared to quantify the total cortical lesion area, and the lesion coverage of different cytoarchitectonic cortical areas.^30,31^

***Brain volume.*** The total brain volume and the cortical volume (mm^3^) were measured from histologic sections. From all 15 rats included in the study, every third section between AP 3.72 mm and -6.60 mm was outlined, and the area (mm^2^) was measured. From the same sections, the cortical area was outlined from the ipsi- and contralateral sides, and the total area (mm^2^) was measured. The brain or cortical area was then multiplied by the section thickness to obtain the volume (mm^3^).

**Supplementary Figure 1.** Photomicrographs showing a dorsal view to the perfusion-fixed brain of **(A)** a rat with a small lateral fluid-percussion injury (FPI) -induced lesion (#93), **(B)** a rat with a large FPI -induced lesion (#108), and **(C)** rat #112. **(D)** Posterior view of rat #112 brain. Arrowheads point to a swollen appearance and transparency of the cortical surface. **(E-H)** Photomicrographs of different coronal planes of rat #112 brain (panel E is the most rostral and panel H the most caudal), which were taken during cutting with a sliding microtome. The brain was embedded in OCT (optimal cutting temperature compound, #23-730-571, Fisher Scientific) and surrounded with dry ice. Note the thinning of slightly curled (panels G-H) cortical tissue and enlarged ventricles (panels E-F), particularly caudally.

**Supplementary Figure 2.** Photographs of a rat with a small lesion (top, #93), a rat with a large lesion (middle, #108), and rat #112 (bottom) at baseline. Rat #112 had a normal physical appearance including skull shape.

**Supplementary video 1.** Rat #112 beam walking at baseline. Beam scores for each trial were: trial 1, score 5; trial 2, score 6; trial 3, score 5. Note: preferably open the video using VLC media player.

**Supplementary video 2.** Rat #112 beam walking at day 30 post-TBI. Beam scores for each trial were: trial 1, score 0; trial 2, score 0; trial 3, score 0. Note: preferably open the video using VLC media player.

**Supplementary video 3.** Rat #112 was monitored on day 11 after TBI. The X-axis shows the time of day (video started at 7:00 a.m.). The colored bars at the bottom indicate the sleep stage. Blue indicates the wake stage. Green indicates the N2 stage. Red indicates K-S. Purple indicates REM. Note that the rat never entered N3 sleep. The Y-axis shows the voltage. EEG channels: C1, ipsilateral frontal; O1, ipsilateral occipital; C2, contralateral frontal; O2, contralateral occipital. Note: preferably open the video using VLC media player.

**Supplementary video 4.** Rat #93 was monitored on day 12 after TBI. The X-axis shows the time of day (video started at 7:00 a.m.). The colored bars at the bottom indicate the sleep stage. Blue indicates the wake stage. Green indicates the N2 stage. Turquoise indicates N3. Purple indicates REM. The Y-axis shows the voltage. EEG channels: C1, ipsilateral frontal; O1, ipsilateral occipital; C2, contralateral frontal; O2, contralateral occipital. Note: preferably open the video using VLC media player.

**REFERENCES**

17. Andrade P, Nissinen J, Pitkänen A. Generalized Seizures after Experimental Traumatic Brain Injury Occur at the Transition from Slow-Wave to Rapid Eye Movement Sleep. JNeurotrauma. 2017;34(7):1482–1487; doi: [10.1089/neu.2016.4675](https://doi.org/10.1089/neu.2016.4675).

26. McIntosh TK, Vink R, Noble L, et al. Traumatic brain injury in the rat: characterization of a lateral fluid-percussion model. Neuroscience. 1989;28(1):233–244; doi: [10.1016/0306-4522(89)90247-9](https://doi.org/10.1016/0306-4522(89)90247-9).

27. Paxinos G, Watson C, (6^th^ ed). The Rat Brain in Stereotaxic Coordinates. Elsevier. Amsterdam, The Netherlands; 2007.

28. Ohlsson AL, Johansson BB. Environment influences functional outcome of cerebral infarction in rats. Stroke. 1995;26(4):644–649; doi: [10.1161/01.str.26.4.644](https://doi.org/10.1161/01.str.26.4.644).

29. Nissinen J, Andrade P, Natunen T, et al. Disease-modifying effect of atipamezole in a model of post-traumatic epilepsy. Epilepsy Res. 2017;136:18–34; doi: [10.1016/j.eplepsyres.2017.07.005](https://doi.org/10.1016/j.eplepsyres.2017.07.005).

30. Ekolle Ndode-Ekane X, Kharatishvili I, Pitkänen A. Unfolded Maps for Quantitative Analysis of Cortical Lesion Location and Extent after Traumatic Brain Injury. JNeurotrauma. 2017;34(2):459–474; doi: [10.1089/neu.2016.4404](https://doi.org/10.1089/neu.2016.4404).

31. Web application for creating two dimensional unfolded maps. Available from: [https://unfoldedmap.org](about:blank) [Last accessed: 12/28/2022]
