# Supplementary material for: Sleep Disturbance and Severe Hydrocephalus in a Normally Behaving Wistar Rat With Traumatic Brain Injury
Source: Neurotrauma Rep. 2023 Jun 19;4(1):384–95. doi: 10.1089/neur.2022.0090 (PMC10282974; doi:10.1089/neur.2022.0090)

**Supplementary Figure 1.** Photomicrographs showing a dorsal view to the perfusion-fixed brain of **(A)** a rat with a small lateral fluid-percussion injury (FPI) -induced lesion (#93), **(B)** a rat with a large FPI -induced lesion (#108), and **(C)** rat #112. **(D)** Posterior view of rat #112 brain. Arrowheads point to a swollen appearance and transparency of the cortical surface. **(E-H)** Photomicrographs of different coronal planes of rat #112 brain (panel E is the most rostral and panel H the most caudal), which were taken during cutting with a sliding microtome. The brain was embedded in OCT (optimal cutting temperature compound, #23-730-571, Fisher Scientific) and surrounded with dry ice. Note the thinning of slightly curled (panels G-H) cortical tissue and enlarged ventricles (panels E-F), particularly caudally.


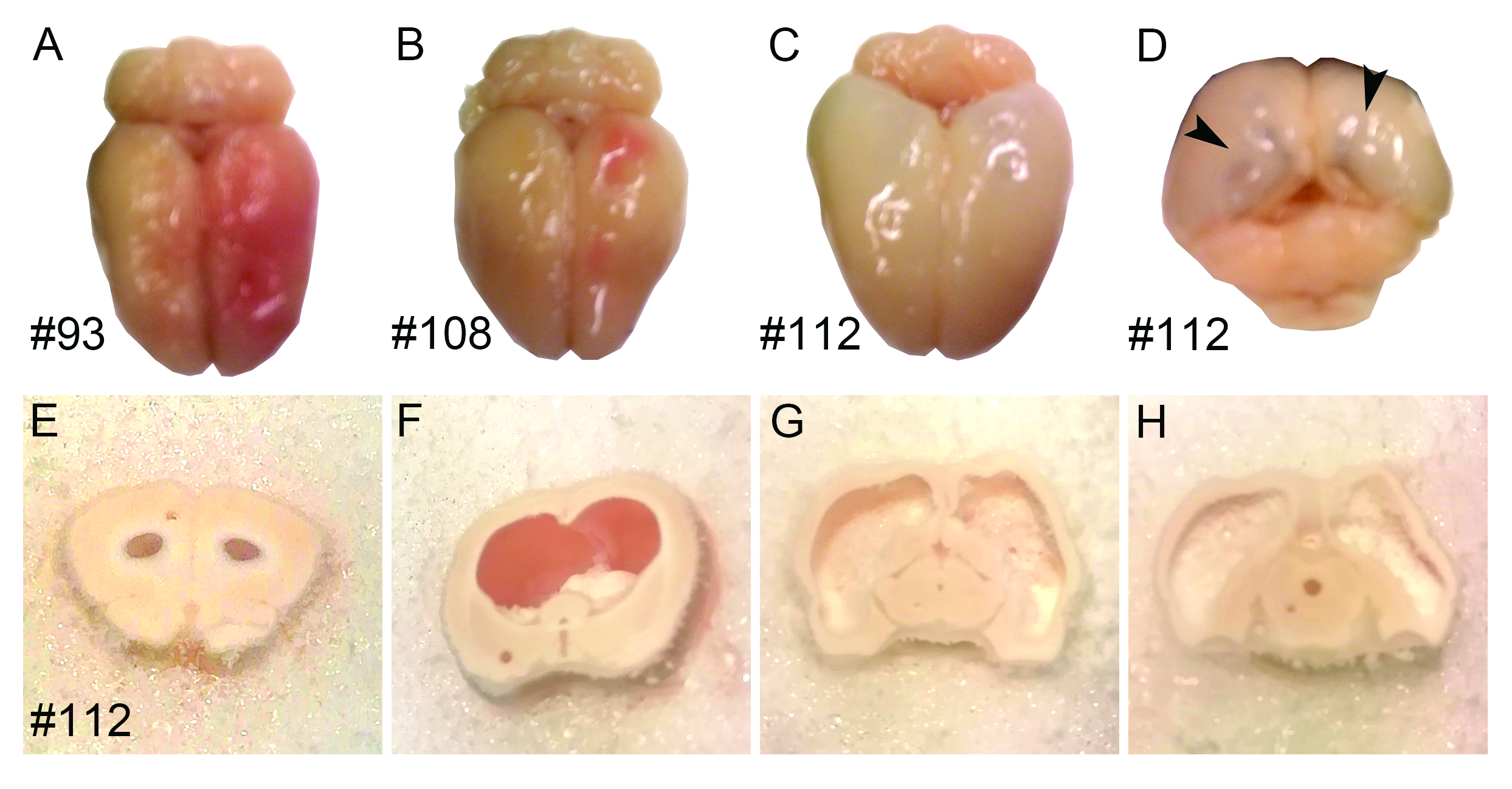

Supplement: Supplemental data [file Suppl_FigS1.docx]
