# Supplementary material for: Sleep Disturbance and Severe Hydrocephalus in a Normally Behaving Wistar Rat With Traumatic Brain Injury
Source: Neurotrauma Rep. 2023 Jun 19;4(1):384–95. doi: 10.1089/neur.2022.0090 (PMC10282974; doi:10.1089/neur.2022.0090)

**Supplementary Figure 2.** Photographs of a rat with a small lesion (top, #93), a rat with a large lesion (middle, #108), and rat #112 (bottom) at baseline. Rat #112 had a normal physical appearance including skull shape.


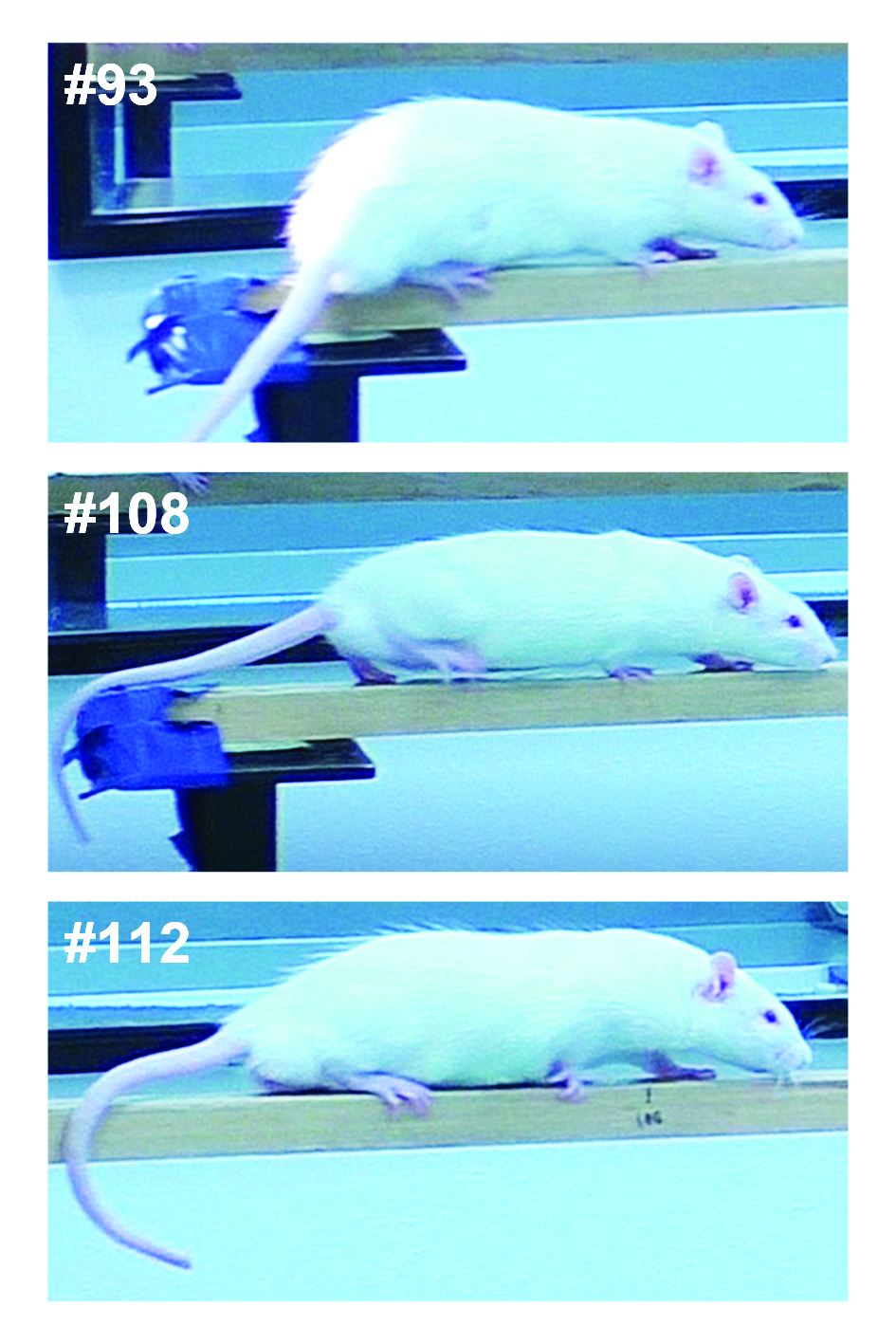

Supplement: Supplemental data [file Suppl_FigS2.docx]
